# Sensor Fault Reconstruction Using Robustly Adaptive Unknown-Input Observers

**DOI:** 10.3390/s24103224

**Published:** 2024-05-19

**Authors:** Qiang Huang, Zhi-Wei Gao, Yuanhong Liu

**Affiliations:** Research Centre for Digitalization and Intelligent Diagnosis to New Energies, College of Electrical and Information Engineering, Northeast Petroleum University, Daqing 163318, China; huang_kai_qiang@163.com (Q.H.); liuyuanhong@nepu.edu.cn (Y.L.)

**Keywords:** sensor fault, fault reconstruction, unknown input uncertainties, linear matrix inequality, Lipschitz nonlinear system, aircraft systems, robotic arm

## Abstract

Sensors are a key component in industrial automation systems. A fault or malfunction in sensors may degrade control system performance. An engineering system model is usually disturbed by input uncertainties, which brings a challenge for monitoring, diagnosis, and control. In this study, a novel estimation technique, called adaptive unknown-input observer, is proposed to simultaneously reconstruct sensor faults as well as system states. Specifically, the unknown input observer is used to decouple partial disturbances, the un-decoupled disturbances are attenuated by the optimization using linear matrix inequalities, and the adaptive technique is explored to track sensor faults. As a result, a robust reconstruction of the sensor fault as well as system states is then achieved. Furthermore, the proposed robustly adaptive fault reconstruction technique is extended to Lipschitz nonlinear systems subjected to sensor faults and unknown input uncertainties. Finally, the effectiveness of the algorithms is demonstrated using an aircraft system model and robotic arm and comparison studies.

## 1. Introduction

Increased sophisticated industrial products put higher requirements on industrial systems, making industrial systems more complex and expensive. The components, such as sensors, are prone to faults. If the faults are not detected at an early stage and appropriate measures are not taken in a timely manner, the system performance will be degraded, and the cost of industrial production will be increased. Therefore, it is important to develop an effective fault diagnosis approach to improve the reliability and safety of industrial systems. Over the past few decades, various diagnostic technologies based on information redundancy have been developed [1,2,3,4,5] and different applications such as in aerospace area and energy systems [6,7,8,9] were reported.

The methods of fault diagnosis can be classified from different perspectives. According to a recent review [10], fault diagnosis technology can be divided into model-based methods, signal-based approaches, knowledge-based techniques, and hybrid methods. If the model is available, model-based fault diagnosis has proved to be a powerful diagnosis technique. A fault detection filter is a well-known diagnosis approach that makes the residual sensitive to faults but insensitive to unknown disturbances by using optimization methods [11,12]. Decoupling technologies, such as the differential geometry method [13,14] and the unknown input observer technique [15,16], are alternatives to remove the effect of the unknown disturbances on residuals to improve the accuracy of fault diagnosis. For the case when unknown input uncertainties cannot be decoupled completely, a fault diagnosis approach was developed in [17] by synthesizing the advantages of optimization technology and decoupling technology.

Fault reconstruction, or fault estimation, is an excellent method for fault diagnosis, which can not only detect and locate faults, but also identify the type, shape, and size of faults, which also can provide useful information for active fault-tolerant control design. The typical fault estimation approaches include the descriptor system method [18], sliding-mode approach [19,20] augmented system method [17,21], proportional-integral approach [22,23,24], and adaptive observer method [25,26,27,28]. All the above approaches have their own merits. It is noted that [17] considered partially decoupled unknown input uncertainties, and proposed a robustly augmented fault estimation technique, which suited more wide engineering scenarios. The augmented UIO observers are a powerful tool for fault reconstruction, but they could have imperfect tracking performance when the fault signal is high-frequent. This motivates us to develop a novel UIO fault estimation technique for dynamic systems under partially decoupled input uncertainties. The innovations and contributions of this study are listed below:

(i) A novel sensor fault estimation approach is proposed by integrating unknown input observer and adaptive observer techniques, which is named as adaptive unknown input observer (UIO) approach.
(ii) An unknown input observer is used to decouple partial input uncertainties, and the linear matrix inequality approach is employed to attenuate un-decoupled unknown input uncertainty and the differential of the sensor fault. As a result, a robust reconstruction of the sensor fault is achieved.
(iii) Without the aid of the well-known augmented system technique, the proposed adaptive observer technique can achieve a direct reconstruction of the sensor fault, which provides a novel way for sensor fault reconstruction.
(iv) The observer gains are solved by using strict linear matrix inequalities without equality constraints, which are more convenient for calculation.
(v) The proposed sensor fault estimation approaches are developed for both linear systems and Lipschitz nonlinear systems, which have a wide applicability.
(vi) The effectiveness of the proposed sensor fault algorithms is validated by two engineering-oriented examples, and comparison studies are carried out to demonstrate the tracing performance of the proposed technique.

In this paper, the following notations are used:

${\left\|•\right\|}_{2}$ represents the 2-norm in Euclidean space; the super-script symbol T stands for the transpose of matrices or vectors; $R^{n}$ and $R^{n\times m}$ denote n-dimensional Euclidean space and the set of n×m real matrices, respectively; 0 stands for scalar zero or a zero matrix with appropriate zero entries; I is an identity matrix with appropriate dimensionality; ${\left\|d\right\|}_{Tf}=(\int_{0}^{Tf}d^{T}(t)d(t)dt{)}^{\frac{1}{2}}$ and $\left[\begin{array}{c} S_{1}\;S_{2}\\ {}*\;S_{3} \end{array}\right]=\left[\begin{array}{c} S_{1}\;S_{2}\\ S_{2}^{T}\;S_{3} \end{array}\right]$.

## 2. Robustly Adaptive Sensor Fault Estimation for Linear System

### 2.1. System Description

Consider a linear system with sensor fault as follows:

(1) $$\left\{\begin{array}{l} \dot{x}\left(t\right)=Ax\left(t\right)+Bu\left(t\right)+B_{d}d\left(t\right)\;\\ y\left(t\right)=Cx\left(t\right)+Df_{s}(t)\;\;\;\;\;\;\;\;\;\;\;\;\;\;\;\;\;\;\; \end{array}\right.$$

where $x(t)\in R^{n}$, $y(t)\in R^{m}$ are the state vector and measurement output vector, respectively. $u\left(t\right)$ represents input vector, $f_{s}\in R^{ls}$ is the time-varying sensor fault vector, $d(t)\in R^{ld}$ is a bounded vector standing for unknown input uncertainty. A, B, C, D and ${\;B}_{d}$ are the known system matrices with appropriate dimensions. For the simplicity of the formula, the symbol of t will be omitted in the rest of this paper.

In addition, the disturbance distribution matrix and disturbance are decomposed as $B_{d}=[B_{d1},B_{d2}]$ and $d={\left[d_{1}^{T},d_{2}^{T}\right]}^{T}$. It is assumed that $d_{1}\in R^{l_{d1}}$ is decoupled while $d_{2}\in R^{l_{d2}}$ cannot be decoupled.

### 2.2. Adaptive UIO Design for Sensor Fault Reconstruction

It is noted that both sensor faults and input uncertainties exist in system (1). A novel unknown input observer is proposed as below:

(2) $$\left\{\begin{array}{l} \dot{z}=Nz+Ly+TBu-L_{1}D{\hat{f}}_{s}\\ \hat{x}=z+My\;\;\;\;\;\;\;\;\;\;\;\;\;\;\;\;\;\;\;\;\;\;\;\;\;\;\;\;\;\;\;\;\;\;\\ \hat{y}=C\hat{x}+D{\hat{f}}_{s}\;\;\;\;\;\;\;\;\;\;\;\;\;\;\;\;\;\;\;\;\;\;\;\;\;\;\;\;\;\; \end{array}\right.$$

where $z\in R^{n}$ is the observer state vector, $\hat{x}\in R^{n}$ and ${\hat{f}}_{s}\in R^{l_{s}}$, respectively, stand for the estimations of system state x and sensor fault $f_{s}$. $N\in R^{n\times n}$, $L=L_{1}+L_{2},\;L_{1}\in R^{n\times m}$, $L_{2}\in R^{n\times m}$, $T\in R^{n\times n}$, $M\in R^{n\times m}$ represent the gains of the observer (2) to be designed.

The estimation errors of the states, sensor fault and output are defined as follows:

(3) $$e_{x}=x-\hat{x}$$

(4) $$e_{s}=f_{s}-{\hat{f}}_{s}$$

(5) $$e_{y}=y-\hat{y}=Ce_{x}+De_{s}$$

The following adaptive law is used to achieve sensor fault estimation:

(6) $${\dot{\hat{f}}}_{s}=\Gamma Fe_{y}$$

where $\Gamma \in R^{l_{s}\times l_{s}}$ is a constant symmetric positive definite matrix, used as the learning rate, and $F\in R^{l_{s}\times m}$ is the gain matrix to be designed.

By using (1)–(3), one has

(7) $$\begin{array}{c} e_{x}=x-\hat{x}\;\;\;\;\;\;\;\;\;\;\;\;\;\;\;\;\;\;\;\;\;\;\;\;\;\;\;\;\;\;\;\;\;\;\\ =x-z-MCx-MDf_{s}\;\;\\ \;\;\;=(I-MC)x-z-MDf_{s}\;\;\; \end{array}$$

Taking the derivative of (7) and using (1) and (2), one can derive the state error dynamic as follows:

(8) $$\begin{array}{c} {\dot{e}}_{x}=\left(I-MC\right)\dot{x}-\dot{z}-MD{\dot{f}}_{s}\;\;\;\;\;\;\;\;\;\;\;\;\;\;\;\;\;\;\;\;\;\;\;\;\;\;\;\;\;\;\;\;\;\;\;\;\;\;\;\;\;\;\;\;\;\;\;\;\;\;\;\;\;\;\;\;\;\;\;\;\;\;\;\;\;\;\;\;\;\;\;\;\;\;\;\;\;\;\\ =\left(I-MC\right)\left[Ax+Bu+B_{d}d\right]-\left(Nz+Ly+TBu-L_{1}D{\hat{f}}_{s}\right)-MD{\dot{f}}_{s}\;\;\;\;\;\;\;\;\;\;\;\;\\ \;\;=\left(I-MC\right)\left[Ax+Bu+B_{d}d\right]-Nz-L_{1}Cx-L_{1}Df_{s}-L_{2}y\;\;\;\;\;\;\;\;\;\;\;\;\;\;\;\;\;\;\;\;\;\;\;\;\;\;\;\;\;\;\;\;\\ \;\;\;\;\;\;\;-TBu+L_{1}D{\hat{f}}_{s}-MD{\dot{f}}_{s}\;\;\;\;\;\;\;\;\;\;\;\;\;\;\;\;\;\;\;\;\;\;\;\;\;\;\;\;\;\;\;\;\;\;\;\;\;\;\;\;\;\;\;\;\;\;\;\;\;\;\;\;\;\;\;\;\;\;\;\;\;\;\;\;\;\;\;\;\;\;\;\;\;\;\;\;\;\;\;\;\;\;\;\;\;\;\;\;\;\;\;\;\;\;\\ \;=\left[\left(I-MC\right)A-L_{1}C\right]e_{x}+\left[\left(I-MC\right)A-L_{1}C-N\right]z\;\;\;\;\;\;\;\;\;\;\;\;\;\;\;\;\;\;\;\;\;\;\;\;\;\;\;\;\;\;\;\;\;\;\;\;\;\;\;\;\\ \;\;+\left\{[(I-MC)A-L_{1}C]M-L_{2}\right\}y+\left(I-MC\right)Bu-TBu\;\;\;\;\;\;\;\;\;\;\;\;\;\;\;\;\;\;\;\;\;\;\;\;\;\;\;\;\;\;\;\;\;\;\;\;\\ +\left(I-MC\right)B_{d1}d_{1}+\left(I-MC\right)B_{d2}d_{2}-MD{\dot{f}}_{s}-L_{1}De_{s}\;\;\;\;\;\;\;\;\;\;\;\;\;\;\;\;\;\;\;\;\;\;\;\;\;\;\;\;\;\;\;\;\;\;\;\;\; \end{array}$$

where $B_{d}=\left[B_{d1}B_{d2}\right]$

To simplify (8), one needs the following conditions:

(9) T=(I−MC)

(10) $$N=(I-MC)A-L_{1}C$$

(11) $$L_{2}=[(I-MC)A-L_{1}C]M$$

(12) $$(I-MC)B_{d1}=0$$

To ensure the solvability of the conditions above, the following assumption is necessary.

**Assumption** **1** ([17,29])**.**

*(a)*  $rank(CB_{d1})=rank(B_{d1})$
*(b)*  *The pair $\left(C,A\right)$ is detectable.*
*(c)*  $rank\left[\begin{array}{c} A-sI\;\;0\\ C\;\;\;\;\;\;\;\;D \end{array}\right]=n+l_{s}$*, for all* s *with Re(s)≥0, but s≠0*.

**Remark** **1.**

*(i)*  *(a) in Assumption 1 can ensure the disturbance* $d_{1}$ *can be completely decoupled, and one can calculate a special solution by *$M=B_{d1}[(CB_{d1}{)}^{T}(CB_{d1}){]}^{-1}(CB_{d1}{)}^{T}$.
*(ii)*  *(b) and (c) in Assumption 1 can ensure one can find an observer gain to make the matrix N stable.*

From (9)–(12), the state estimation error dynamic (8) becomes:

(13) $$\begin{array}{c} {\dot{e}}_{x}=\left(I-MC\right)\dot{x}-\dot{z}-MD{\dot{f}}_{s}\;\;\;\;\;\;\;\;\;\\ =Ne_{x}-L_{1}De_{s}+TB_{d2}d_{2}-MD{\dot{f}}_{s} \end{array}$$

Using (4)–(6), the following sensor fault error dynamic can be obtained

(14) $${\dot{e}}_{s}={\dot{f}}_{s}-\Gamma FCe_{x}-\Gamma FDe_{s}$$

For subsequent derivation, the following lemmas are useful and necessary.

**Lemma** **1**  [30]**.** *For any scalar ε>0* *and given matrices of H, J* *and a time-varying matric S(t)* *with $\left\|s(t)\right\|<1$, we have:*

$$HS(t)J+J^{T}S(t)H^{T}\leq {\varepsilon^{-1}H}^{T}H+\varepsilon J^{T}J$$

**Lemma** **2** [31]**.** *For a symmetric matrix $Q=\left[\begin{array}{c} Q_{11}\;Q_{12}\\ \;*\;\;\;Q_{22} \end{array}\right]$, Q<0* *is equivalent $Q_{22}<0$* *and $Q_{11}-Q_{12}Q_{22}^{-1}Q_{12}^{T}<0$*.

It is time to give our main result.

**Theorem** **1.** *The error dynamic Equations (13) and (14) are robustly stale and satisfy ${\left\|e\right\|}_{T_{f}}\leq {r^{2}\left\|d_{f}\right\|}_{T_{f}}$, if there exists a symmetric positive definite matrix $P\in R^{n\times n}$and matrices ${\hat{L}}_{1}\in R^{n\times m}\;$, $F\in R^{l_{s}\times m}$, and scalar r* *such that*

(15) $$\left[\begin{array}{cccc} PTA+A^{T}T^{T}P-{\hat{L}}_{1}C-C^{T}{\hat{L}}_{1}^{T}+I & -{\hat{L}}_{1}D-(FC{)}^{T} & -PMD & PTB_{d2}\\ {}* & -FD-{\left(FD\right)}^{T}+I\; & {\;\;\Gamma }^{-1} & 0\\ {}* & * & -r^{2}I & 0\\ {}* & * & * & -r^{2}I \end{array}\right]<0$$

*where r* *is a performance index that represents the magnitude of error compared with disturbance and $e={\left[e_{x}^{T}e_{s}^{T}\right]}^{T}$, $d_{f}={\left[{\dot{f}}_{s}^{T}d_{2}^{T}\right]}^{T}.$The gain can be calculated via ${L_{1}=P^{-1}\hat{L}}_{1}$*.

**Proof** **of** **Theorem** **1.**  Choosing the following Lyapunov function candidate of the error dynamic system (13) and (14):

(16) $$V\left(e_{x},e_{s}\right)=e_{x}^{T}Pe_{x}+e_{s}^{T}{\Gamma^{-1}e}_{s}$$

Taking the derivative of (16) and using (13)–(14), it is derived that

(17) $$\begin{array}{c} \dot{V}\left(e_{x},e_{s}\right)={\dot{e}}_{x}^{T}Pe_{x}+e_{x}^{T}P{\dot{e}}_{x}+e_{s}^{T}\Gamma^{-1}{\dot{e}}_{s}+{\dot{e}}_{s}^{T}{\Gamma^{-1}e}_{s}\;\;\;\;\;\;\;\;\;\;\;\;\;\;\;\;\;\;\;\;\;\;\;\;\;\;\;\;\;\;\;\;\;\;\;\;\;\;\;\;\;\;\;\;\;\;\;\;\;\;\;\;\;\;\;\;\;\;\;\;\;\;\;\;\\ =e_{x}^{T}\left(PN+N^{T}P\right)e_{x}-2e_{x}^{T}PL_{1}De_{s}+2e_{x}^{T}PTB_{d2}d_{2}-2e_{x}^{T}PMD{\dot{f}}_{s}+e_{s}^{T}\Gamma^{-1}{\dot{e}}_{s}+{\dot{e}}_{s}^{T}\Gamma^{-1}e_{s}\;\;\;\\ =e_{x}^{T}(PN+N^{T}P)e_{x}-2e_{x}^{T}PL_{1}De_{s}+2e_{x}^{T}PTB_{d2}d_{2}-2e_{x}^{T}PMD{\dot{f}}_{s}+2e_{s}^{T}\Gamma^{-1}{\dot{f}}_{s}-2e_{s}^{T}FCe_{x}\\ \;\;\;\;\;\;\;\;\;\;\;\;\;\;\;\;\;\;\;+e_{s}^{T}\left[-FD-{\left(FD\right)}^{T}\right]e_{s}\;\;\;\;\;\;\;\;\;\;\;\;\;\;\;\;\;\;\;\;\;\;\;\;\;\;\;\;\;\;\;\;\;\;\;\;\;\;\;\;\;\;\;\;\;\;\;\;\;\;\;\;\;\;\;\;\;\;\;\;\;\;\;\;\;\;\;\;\;\;\;\;\;\;\;\;\;\;\;\;\;\;\;\;\;\;\;\;\;\;\;\;\;\;\;\;\;\;\;\;\;\;\;\;\;\;\;\;\;\;\;\;\;\;\;\;\;\;\;\;\;\;\;\\ \;\;\;=e_{x}^{T}\left(PN+N^{T}P\right)e_{x}-2e_{x}^{T}\left[PL_{1}D+(FC{)}^{T}\right]e_{s}+e_{s}^{T}\left[-FD-{\left(FD\right)}^{T}\right]e_{s}+2e_{x}^{T}PTB_{d2}d_{2\;\;\;}\;\;\;\;\\ \;\;\;\;\;\;\;\;\;\;\;\;\;\;\;\;\;\;\;\;-2e_{x}^{T}PMD{\dot{f}}_{s}+2e_{s}^{T}(\Gamma^{-1}){\dot{f}}_{s}\;\;\;\;\;\;\;\;\;\;\;\;\;\;\;\;\;\;\;\;\;\;\;\;\;\;\;\;\;\;\;\;\;\;\;\;\;\;\;\;\;\;\;\;\;\;\;\;\;\;\;\;\;\;\;\;\;\;\;\;\;\;\;\;\;\;\;\;\;\;\;\;\;\;\;\;\;\;\;\;\;\;\;\;\;\;\;\;\;\;\;\;\;\;\;\;\;\;\;\;\;\;\;\;\;\;\;\;\;\;\;\;\;\;\;\;\;\\ \;\;\;\;\;\;\;\;\;\;\;\;\;=\left[\;e_{x}^{T}e_{s}^{T}\right]\Delta \left[\begin{array}{c} e_{x}\\ e_{s} \end{array}\right]+2e_{x}^{T}{PTB}_{d2}d_{2}-2e_{x}^{T}PMD{\dot{f}}_{s}+2e_{s}^{T}\Gamma^{-1}{\dot{f}}_{s}\;\;\;\;\;\;\;\;\;\;\;\;\;\;\;\;\;\;\;\;\;\;\;\;\;\;\;\;\;\;\;\;\;\;\;\;\;\;\;\;\;\;\;\;\;\;\;\;\;\;\;\;\;\;\;\;\;\;\;\;\;\;\; \end{array}$$

where

(18) $$\Delta =\left[\begin{array}{c} PN+N^{T}P\;\;-PL_{1}D-(FC{)}^{T}\\ {}*\;\;\;\;\;\;\;\;\;\;\;\;\;\;\;\;-FD-(FD{)}^{T} \end{array}\right]$$

It is noted that ${\hat{L}}_{1}=PL_{1}$ and $N=TA-L_{1}C.\;$ Therefore, we have:

(19) $$PTA+A^{T}T^{T}P-{\hat{L}}_{1}C-C^{T}{\hat{L}}_{1}^{T}+I=PN+N^{T}P+I$$

The first two-row and two-column matrix of (15) can be thus written as

(20) $${Ω}_{12}=\left[\begin{array}{cc} PN+N^{T}P+I & -PL_{1}D-(FC{)}^{T}\\ {}* & \;\;\;\;-FD-{\left(FD\right)}^{T}+I\; \end{array}\right]$$

From (15) one can know ${Ω}_{12}<0$ in (20) indicating Δ<0 in (18). Therefore from (17), one has $\dot{V}\left(\left(e_{x},e_{s}\right)\right)<0$ when $d_{2}=0$ and ${\dot{f}}_{s}=0$. It implies the estimation error dynamics (13) and (14) are asymptotically stable when $d_{2}=0$ and ${\dot{f}}_{s}=0.$

Then, it is time to consider the scenario when $d_{2}\neq 0$ and ${\dot{f}}_{s}\neq 0$.

Define

(21) $$\begin{array}{c} ϒ=\int_{0}^{Tf}\left(e_{x}^{T}e_{x}+e_{s}^{T}e_{s}-r^{2}d_{f}^{T}d_{f}\right)dt\;\;\;\;\;\;\;\;\;\;\;\;\;\;\;\;\;\;\;\;\;\;\;\;\;\;\;\;\;\;\;\;\;\;\;\;\;\;\;\;\;\;\;\;\;\;\;\;\;\;\;\;\;\;\;\;\;\;\;\;\;\;\;\\ =\int_{0}^{Tf}\left(\dot{V}(e_{x},e_{s})+e_{x}^{T}e_{x}+e_{s}^{T}e_{s}-r^{2}d_{f}^{T}d_{f}\right)dt-\int_{0}^{Tf}\dot{V}(e_{x},e_{s})dt\;\;\;\;\;\;\;\;\;\;\; \end{array}$$

Using (17) and (21), one has

(22) $$\begin{array}{c} ϒ=\int_{0}^{Tf}\left(\dot{V}\left(e_{x},e_{s}\right)+e_{x}^{T}e_{x}+e_{s}^{T}e_{s}-r^{2}d_{f}^{T}d_{f}\right)dt-\int_{0}^{Tf}\dot{V}\left(e_{x},e_{s}\right)dt\;\;\;\;\;\;\;\;\;\;\;\;\;\;\;\;\;\;\;\;\;\;\;\;\;\;\;\;\;\;\;\;\;\;\;\;\;\;\;\;\;\;\\ =\int_{0}^{Tf}\left\{e_{x}^{T}(PN+N^{T}P)e_{x}\right.-2e_{x}^{T}\left[\left(PL_{1}D\right)+(FC{)}^{T}\right]e_{s}+e_{x}^{T}e_{x}+2e_{x}^{T}PTB_{d2}d_{2}-2e_{x}^{T}PMD{\dot{f}}_{s}\\ \;\;+2e_{s}^{T}\Gamma^{-1}{\dot{f}}_{s}+e_{s}^{T}\left[-FD-{\left(FD\right)}^{T}\right]e_{s}+e_{s}^{T}e_{s}-r^{2}{\dot{f}}_{s}^{T}{\dot{f}}_{s}\left.-r^{2}d_{2}^{T}d_{2}\right\}dt-\int_{0}^{Tf}\dot{V}\left(e_{x},e_{s}\right)dt\;\;\;\;\;\;\;\;\;\\ =\int_{0}^{Tf}\left[e_{x}^{T}\;e_{s}^{T}\;{\dot{f}}_{s}^{T}\;d_{2}^{T}\right]\Xi \left[\begin{array}{c} e_{x}\\ e_{s}\\ {\dot{f}}_{s}\\ \;d_{2} \end{array}\right]dt-\int_{0}^{Tf}\dot{V}\left(e_{x},e_{s}\right)dt\;\;\;\;\;\;\;\;\;\;\;\;\;\;\;\;\;\;\;\;\;\;\;\;\;\;\;\;\;\;\;\;\;\;\;\;\;\;\;\;\;\;\;\;\;\;\;\;\;\;\;\;\;\;\;\;\;\;\;\;\;\;\;\;\; \end{array}$$

where

(23) $$\Xi =\left[\begin{array}{cccc} PN+N^{T}P+I\;\; & -PL_{1}D-(FC{)}^{T} & -PMD & PTB_{d2}\\ {}* & -FD-{\left(FD\right)}^{T}+I & \Gamma^{-1} & 0\\ {}* & * & -r^{2}I & 0\\ {}* & * & * & -r^{2}\;I \end{array}\right]$$

Noting ${\hat{L}}_{1}=PL_{1}$ and (19) holds. Therefore, it is clear that (23) is equivalent to the left-hand side of (15), implying Ξ<0. Since $\int_{0}^{Tf}\dot{V}\left(e_{x},e_{s}\right)d\left(t\right)\geq 0$, therefore from (22), one has ϒ≤0 which indicates ${\left\|e\right\|}_{Tf}\leq \gamma^{2}{\left\|d_{f}\right\|}_{T_{f}}$. Therefore, Theorem 1 is proved. □

## 3. Adaptive UIO for Sensor Fault Estimation in Lipschitz Nonlinear System

In this section, we will extend the approach of Section 2 to Lipschitz nonlinear systems. The Lipschitz nonlinear system with unknown input uncertainties and sensor additive fault is represented as follows:

(24) $$\left\{\begin{array}{l} \dot{x}\left(t\right)=Ax\left(t\right)+Bu\left(t\right)+B_{d}d\left(t\right)+\psi (t,x)\\ y\left(t\right)=Cx\left(t\right)+Df_{s}(t)\;\;\;\;\;\;\;\;\;\;\;\;\;\;\;\;\;\;\;\;\;\;\;\;\;\;\;\;\;\;\;\;\;\;\;\;\;\;\;\;\;\;\;\; \end{array}\right.$$

where ψ(t,x) is a nonlinear vector function satisfying the following inequality relationship, and the definitions of the other symbols are the same as defined in (1).

(25) $$\begin{array}{c} \left\|\psi (t,x)\right\|\leq \theta \|x\|\\ \left\|\psi \left(t,x\right)-\psi \left(t,\hat{x}\right)\right\|\leq \theta \left\|x-\hat{x}\right\|\\ \forall \left(t,x\right)\;\mathrm{and}\;\forall (t,\hat{x})\in R\times R^{n} \end{array}$$

Then, a nonlinear adaptive unknown input observer for (24) is given as follows:

(26) $$\left\{\begin{array}{l} \dot{z}=Nz+Ly+TBu-L_{1}D{\hat{f}}_{s}+T\hat{\psi }\\ \hat{x}=z+My\\ \hat{y}=C\hat{x}+D{\hat{f}}_{s}\;\;\;\;\;\;\;\;\;\;\;\;\;\;\;\;\;\;\;\;\;\;\;\;\;\;\;\;\;\;\;\;\;\;\;\;\;\;\; \end{array}\right.$$

in which $\hat{\psi }=\psi (t,\hat{x})$.

In terms of (3), (4) and (22)–(24), one has:

(27) $$\begin{array}{cc} {\dot{e}}_{x} & =\left(I-MC\right)\dot{x}-\dot{z}-MD{\dot{f}}_{s}\\ & =\left(I-MC\right)\left[Ax+Bu+B_{d}d+\psi \right]-\left(Nz+Ly+TBu-L_{1}D{\hat{f}}_{s}+T\hat{\psi }\right)-MD{\dot{f}}_{s}\\ & =\left[\left(I-MC\right)A-L_{1}C\right]e_{x}+\left[\left(I-MC\right)A-L_{1}C-N\right]z\\ & +\left\{[(I-MC)A-L_{1}C]M-L_{2}\right\}y+\left(I-MC\right)Bu-TBu+\left(I-MC\right)\psi -T\hat{\psi }\\ & +\left(I-MC\right)B_{d1}d_{1}+\left(I-MC\right)B_{d2}d_{2}-MD{\dot{f}}_{s}-L_{1}De_{s} \end{array}$$

Letting $\tilde{\psi }=\psi -\hat{\psi }$ and substituting (9)–(12) into (27), one has

(28) $${\dot{e}}_{x}=Ne_{x}-L_{1}De_{s}+TB_{d2}d_{2}+T\tilde{\psi }-MD{\dot{f}}_{s}$$

From (6), one has

(29) $${\dot{e}}_{s}={\dot{f}}_{s}-\Gamma FCe_{x}-\Gamma FDe_{s}$$

**Theorem** **2.** *A robust adaptive unknown input observer for Lipschitz nonlinear system (24) can be established satisfying ${\left\|e\right\|}_{T_{f}}\leq r^{2}{\left\|d_{f}\right\|}_{T_{f}}\;$, if there exists a symmetric positive definite matrix $P\in R^{n\times n}$* *and matrices ${\hat{L}}_{1}\in R^{n\times m}\;$, $F\in R^{l_{s}\times m}$* *and scalars r>0* *and ε>0, such that*

(30) $$\left[\begin{array}{ccccc} PTA+A^{T}T^{T}P-{\hat{L}}_{1}C-C^{T}{\hat{L}}_{1}^{T}+\left(\varepsilon \theta^{2}+1\right)I & -{\hat{L}}_{1}D-(FC{)}^{T} & -PMD & PTB_{d2} & PT\\ {}* & -FD-{\left(FD\right)}^{T}+I & {\;\Gamma }^{-1} & 0 & 0\\ {}* & * & -r^{2}I & 0 & 0\\ {}* & * & * & -r^{2}I & 0\\ {}* & * & * & * & -\varepsilon I \end{array}\right]<0$$

*where r is a performance index and *θ *is Lipschitz constant. The gain can be calculated via* ${L_{1}=P^{-1}\hat{L}}_{1}$.

**Proof** **of** **Theorem** **2.** Choosing the same Lyapunov function candidate defined in (16) and using (28) and (29), one has

(31) $$\begin{array}{l} \dot{V}\left(e_{x},e_{s}\right)=e_{x}^{T}\left(PN+N^{T}P\right)e_{x}-2e_{x}^{T}PL_{1}De_{s}+2e_{x}^{T}PTB_{d2}d_{2}-2e_{x}^{T}PMD{\dot{f}}_{s}\;\\ +2e_{x}^{T}PT\tilde{\psi }+{\dot{e}}_{s}^{T}\Gamma^{-1}e_{s}+e_{s}^{T}\Gamma^{-1}{\dot{e}}_{s}\;\;\;\;\;\;\;\;\;\;\;\;\;\;\;\;\;\;\;\;\;\;\;\;\;\;\;\;\;\;\;\;\;\;\;\;\;\;\;\;\;\;\;\;\;\;\;\;\;\;\;\;\;\;\;\;\;\;\;\;\;\;\;\;\;\;\;\;\;\;\;\;\;\;\;\;\;\;\;\\ =e_{x}^{T}(PN+N^{T}P)e_{x}-2e_{x}^{T}\left[PL_{1}D+(FC{)}^{T}\right]e_{s}+2e_{x}^{T}PTB_{d2}d_{2}-2e_{x}^{T}PMD{\dot{f}}_{s}\\ +2e_{s}^{T}\Gamma^{-1}{\dot{f}}_{s}+e_{s}^{T}\left[-FD-{\left(FD\right)}^{T}\right]e_{s}+2e_{x}^{T}PT\tilde{\psi }\;\;\;\;\;\;\;\;\;\;\;\;\;\;\;\;\;\;\;\;\;\;\;\;\;\;\;\;\;\;\;\;\;\;\;\;\;\;\;\;\;\;\;\;\;\;\;\;\;\;\;\;\;\; \end{array}$$

By applying Lemma 1 to the last term in (31), and using (25), one has

(32) $$\begin{array}{c} \dot{V}(e_{x},e_{s})\leq e_{x}^{T}(PN+N^{T}P)e_{x}-2e_{x}^{T}\left[PL_{1}D+(FC{)}^{T}\right]e_{s}+2e_{x}^{T}PTB_{d2}d_{2}-2e_{x}^{T}PMD{\dot{f}}_{s}\\ +2e_{s}^{T}\Gamma^{-1}{\dot{f}}_{s}+e_{s}^{T}\left[-FD-{\left(FD\right)}^{T}\right]e_{s}+\varepsilon^{-1}e_{x}^{T}PT(PT{)}^{T}e_{x}+\varepsilon {\theta^{2}e}_{x}^{T}e_{x}\;\;\;\;\\ =\left[\;e_{x}^{T}e_{s}^{T}\right]\Pi \left[\begin{array}{c} e_{x}\\ e_{s} \end{array}\right]-2e_{x}^{T}PMD{\dot{f}}_{s}+2e_{x}^{T}PTB_{d2}d_{2}+2e_{s}^{T}\Gamma^{-1}{\dot{f}}_{s}\;\;\;\;\;\;\;\;\;\;\;\; \end{array}$$

in which

(33) $$\;\Pi =\left[\begin{array}{cc} PN+N^{T}P+\varepsilon \theta^{2}I+\varepsilon^{-1}PT(PT{)}^{T} & -PL_{1}D-(FC{)}^{T}\\ {}* & -FD-(FD{)}^{T}\; \end{array}\right]$$

Noting that ${\hat{L}}_{1}=PL_{1}$, and from (19) and (30), one can clearly see Π<0 in (33). As a result, the estimation error dynamics (28) and (29) are asymptotically stable when $d_{2}=0$ and ${\dot{f}}_{s}=0$.

Let

(34) $${ϒ}_{2}=\int_{0}^{Tf}\left(\dot{V}(e_{x},e_{s})+e_{x}^{T}e_{x}+e_{s}^{T}e_{s}-r^{2}d_{f}^{T}d_{f}\right)dt-\int_{0}^{Tf}\dot{V}(e_{x},e_{s})dt$$

Using (32), one has

(35) $$\begin{array}{cc} {ϒ}_{2} & =\int_{0}^{Tf}\left\{e_{x}^{T}\left(PN+N^{T}P+\varepsilon^{-1}PT(PT{)}^{T}+\varepsilon \theta^{2}I+I\right)e_{x}-2e_{x}^{T}\left[PL_{1}D+(FC{)}^{T}\right]e_{s}\right.\\ & +2e_{x}^{T}PTB_{d2}d_{2}-2e_{x}^{T}PMD{\dot{f}}_{s}+2e_{s}^{T}\Gamma^{-1}{\dot{f}}_{s}+e_{s}^{T}\left[-FD-{\left(FD\right)}^{T}+I\right]e_{s}-r^{2}{\dot{f}}_{s}^{T}{\dot{f}}_{s}\left.-r^{2}d_{2}^{T}d_{2}\right\}dt\\ & -\int_{0}^{Tf}\dot{V}(e_{x},e_{s})dt\\ & =\int_{0}^{Tf}\left[\;e_{x}^{T}e_{s}^{T}\dot{f_{s}^{T}}d_{2}^{T}\right]\Psi \left[\begin{array}{c} e_{x}\\ e_{s}\\ {\dot{f}}_{s}\\ \;d_{2} \end{array}\right]dt-\int_{0}^{Tf}\dot{V}\left(e_{x},e_{s}\right)dt \end{array}$$

where

(36) $$\;\Psi =\left[\begin{array}{cccc} PN+N^{T}P+\left(\varepsilon \theta^{2}+1\right)I+\varepsilon^{-1}PT{\left(PT\right)}^{T} & -PL_{1}D-(FC{)}^{T} & -PMD & PTB_{d2}\\ {}* & -FD-{\left(FD\right)}^{T}+I & \Gamma^{-1} & 0\\ {}* & * & -r^{2}I & 0\\ {}* & \;\;* & * & -r^{2}I\; \end{array}\right]\;\;\;$$

Based on (19) and the Schur complement theory in Lemma 2, one can conclude (30) is equivalent to Ψ<0 in (36). It is also noted that $\int_{0}^{Tf}\dot{V}\left(e_{x},e_{f}\right)d\left(t\right)\geq 0$, therefore from (35), one has ${ϒ}_{2}\leq 0$ which indicates ${\left\|e\right\|}_{Tf}\leq r^{2}{\left\|d_{f}\right\|}_{T_{f}}$. We can conclude the estimator error dynamics (28) and (29) are robustly stable. Therefore, Theorem 2 is proved. □

## 4. Design Procedures for Sensor Fault Estimation

The design procedures of the proposed adaptive sensor fault estimators in Section 2 and Section 3 can be summarized as follows:

### 4.1. Procedure 1. Sensor Fault Estimation for Linear Dynamic Systems

a. Select a matrix M as follows:

$$M=B_{d1}[(CB_{d1}{)}^{T}(CB_{d1}){]}^{-1}(CB_{d1}{)}^{T}$$

b. Select the adaptive learning rate Γ, which is a positive definite matrix.
c. Solve the LMI (15) in Theorem 1 to obtain appropriate matrices P, and ${\hat{L}}_{1}$, F, and calculate the estimator gain by $L_{1}=P^{-1}{\hat{L}}_{1}$.
d. Calculate the other estimator gain matrices:

T=I−MC

$$N=(I-MC)A-L_{1}C$$

$$L_{2}=[(I-MC)A-L_{1}C]M$$

e. Implement the robust adaptive estimator in the form of (2) and (6), and the real-time estimate of state $\hat{x}$ and sensor fault ${\hat{f}}_{s}$ can be obtained.

### 4.2. Procedure 2. Sensor Fault Estimation for Lipschitz Nonlinear Dynamic Systems

a. Select a matrix M as follows:

$$M=B_{d1}[(CB_{d1}{)}^{T}(CB_{d1}){]}^{-1}(CB_{d1}{)}^{T}$$

b. Select the adaptive learning rate Γ, which is a positive definite matrix.
c. Solve the LMI (30) in Theorem 2 to obtain appropriate matrices P, and ${\hat{L}}_{1}$, F, and calculate the estimator gain by $L_{1}=P^{-1}{\hat{L}}_{1}$.
d. Calculate the other estimator gain matrices:

T=I−MC

$$N=(I-MC)A-L_{1}C$$

$$L_{2}=[(I-MC)A-L_{1}C]M$$

e. Implement the nonlinear adaptive estimator in the form of (6) and (26), and the real-time estimate of state $\hat{x}$ and sensor fault ${\hat{f}}_{s}$ can be obtained.

All the design procedures above are off-line design, but real-time implementation. The real-time input and output data are used when the observers are implemented.

## 5. Simulation Studies

### 5.1. Civil Aircraft

In this section, a civil aircraft model [32] is used to demonstrate the proposed adaptive UIO technique. Considering sensor fault and partially decoupled input disturbance, the aircraft model is described as follows:

(37) $$\left\{\begin{array}{l} \dot{x}\left(t\right)=Ax\left(t\right)+Bu\left(t\right)+B_{d}d\left(t\right)\;\\ y\left(t\right)=Cx\left(t\right)+Df_{s}(t)\;\;\;\;\;\;\;\;\;\;\;\;\;\;\;\;\;\;\; \end{array}\right.$$

where the input vector $u={\left[u_{1}\;u_{2}\right]}^{T}$ is composed of the elevator deflection angle $\theta_{e}$ and thrust T, and the state vector $x={\left[qV_{tas}\alpha \;\theta \right]}^{T}$ includes the pitch rate, true air speed, angle of attack and pitch angle. The matrices of system (37) are as follows:

(38) $$A=\left[\begin{array}{cccc} 0.6803 & 0.0002 & -1.0490 & 0\\ -0.1463 & -0.0062 & -4.6726 & -9.7942\\ 1.0050 & -0.0006 & -0.5717 & 0\\ 1.0000 & 0 & 0 & 0 \end{array}\right],B=\left[\begin{array}{cc} -1.5539 & 0.0154\\ 0 & 1.3287\\ -0.0398 & -0.0007\\ 0 & 0 \end{array}\right],\;B_{d}=\left[B_{d_{1}}\;\;B_{d_{2}}\right]=\left[\begin{array}{l} \;\;1\;\;\;\;\;\;0.4\\ -1\;\;\;\;\;0.6\\ \;\;1\;\;\;\;\;\;\;0\\ \;\;1\;\;\;-0.4 \end{array}\right],\;C=\left[\begin{array}{l} 1\;\;\;0\;\;\;0\;\;\;0\\ 0\;\;\;1\;\;\;0\;\;\;0\;\;\;\\ 0\;\;\;0\;\;\;0\;\;\;1 \end{array}\right],D=\left[\begin{array}{c} 0\\ 0\\ 1 \end{array}\right].$$

The unknown input disturbances are $d_{1}=\sin(10t)$, $d_{2}=\sin(20t)$. The input signals are $u_{1}=2\sin(\pi t)$ and $u_{2}=2$ and the initial state vector is $x\left(0\right)={\left[0.1-5-0.1\;0.1\right]}^{T}$. The sensor fault is assumed to be:

(39) $$f_{s}=\left\{\begin{array}{cc} 0 & t<5\\ 0.2(t-5) & 5\leq t<10\\ \sin(2t) & t\geq 10\; \end{array}\right.$$

Selecting r=0.12 and Γ=10, using procedure 1, we can obtain the following estimator parameters:

$$M=\left[\begin{array}{ccc} 0.3333 & -0.3333 & 0.3333\\ -0.3333 & 0.3333 & -0.3333\\ 0.3333 & -0.3333 & 0.3333\\ 0.3333 & -0.3333 & 0.3333 \end{array}\right],\;\;T=\left[\begin{array}{cccc} 0.6667 & 0.3333 & 0 & -0.3333\\ 0.3333 & 0.6667 & 0 & 0.3333\\ -0.3333 & 0.3333 & 1 & -0.3333\\ -0.3333 & 0.3333 & 0 & 0.6667 \end{array}\right],$$

$$L_{1}=\left[\begin{array}{ccc} 3.6706 & -0.1416 & -0.7720\\ -0.1834 & 2.9668 & 0.0010\\ 2.2772 & -1.2722 & -0.4654\\ 0.8820 & -2.4025 & -0.1584 \end{array}\right]\times 10^{3},\;L_{2}=\left[\begin{array}{ccc} -1.0154 & 1.0154 & -1.0154\\ 1.0463 & -1.0463 & \;1.0463\\ -1.0293 & \;1.0293 & -1.0293\\ -1.0431 & 1.0431 & -1.0431 \end{array}\right]\times 10^{3},$$

$$L=L_{1}+L_{2}=\left[\begin{array}{ccc} \;2.6552 & 0.8738 & -1.7874\\ 0.8629 & 1.9205 & 1.0473\\ 1.2479 & -0.2429 & -1.4947\\ -0.1611 & -1.3594 & -1.2016 \end{array}\right]\times 10^{3},$$

$$N=\left[\begin{array}{cccc} -3.6714 & 0.1416 & -0.0023 & 0.7687\\ 0.1834 & -2.9668 & -0.0035 & -0.0075\\ -2.2764 & 1.2722 & -0.0018 & 0.4621\\ -0.8812 & 2.4025 & -0.0012 & 0.1552 \end{array}\right]\times 10^{3},$$

$$F=\left[-0.6781\;2.5525\;\;2.5825\right]\times 10^{5}$$

**Figure 1:**
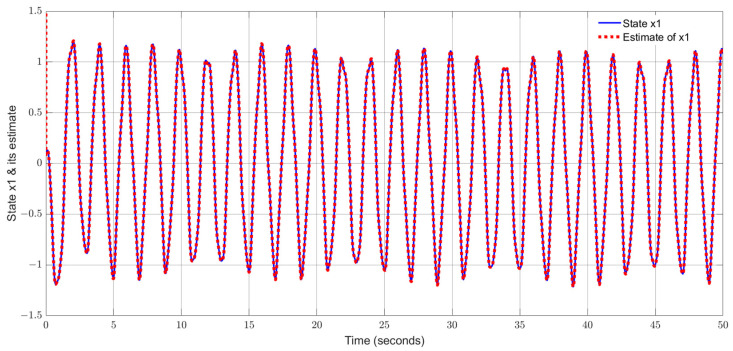
State $x_{1}$ (pitch rate) and its estimate.

**Figure 2:**
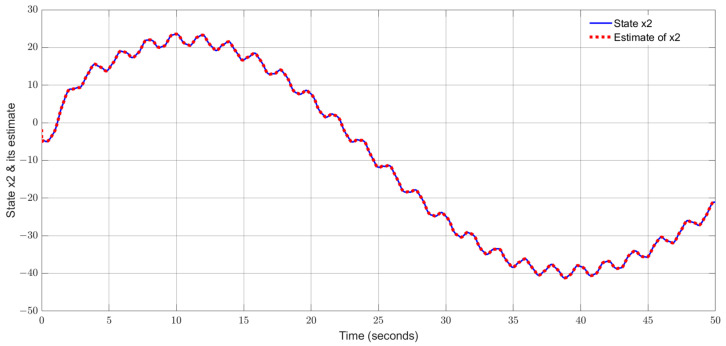
State $x_{2}$ (true airspeed) and its estimate.

**Figure 3:**
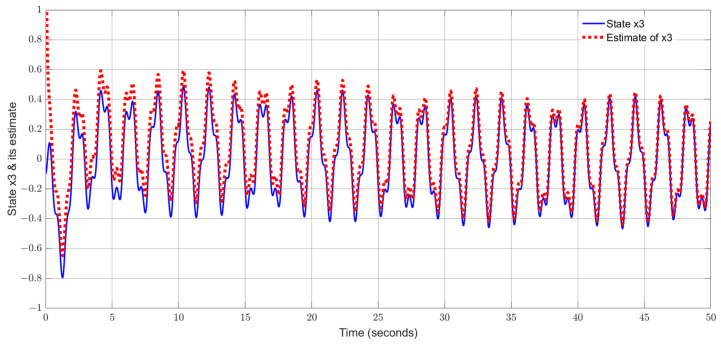
State $x_{3}\;$(attack angle) and its estimate.

**Figure 4:**
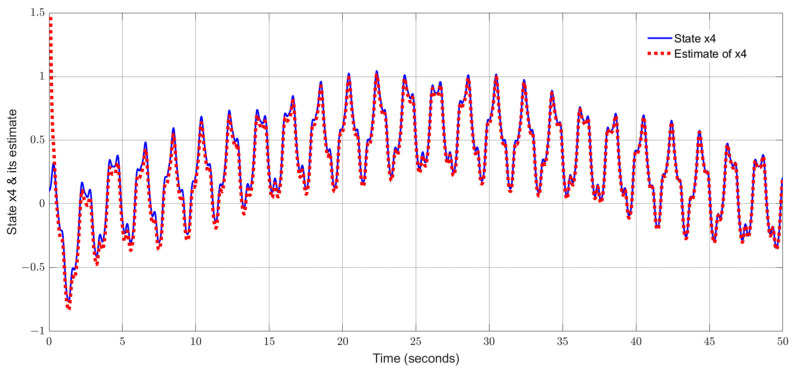
State $x_{4}$ (pitch angle) and its estimate.

**Figure 5:**
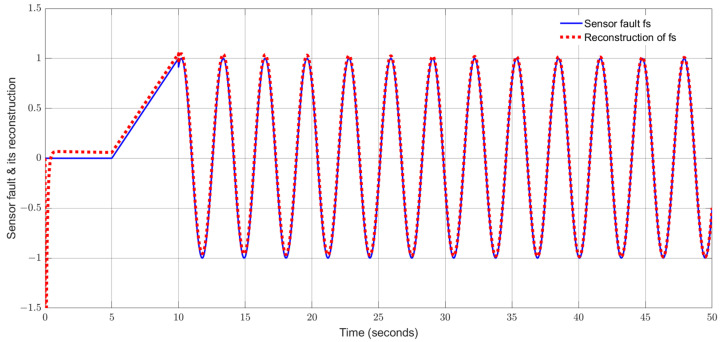
Sensor fault and its reconstruction.

By using the observer gains above and implement the robust adaptive UIO in the form of (2) and (6). Figure 1, Figure 2, Figure 3, Figure 4 show the trajectories of four states and their estimates, which demonstrate the estimated curves can track the real states successfully. Figure 5 depicts the sensor fault $f_{s}(t)$ and its estimate. One can see the estimated signal can well track the sensor fault signal.

### 5.2. Single-Link Flexible Joint Robot

A flexible joint robot linkage manipulator driven by DC motor can be built as a Lipschitz nonlinear system model described as follows [33]:

(40) $$\left\{\begin{array}{l} {\dot{\theta }}_{m}=w_{m}\\ w_{m}=\frac{k}{J_{m}}(\theta_{l}-\theta_{m})-\frac{G}{J_{m}}w_{m}+\frac{k_{\tau }}{J_{m}}u\\ {\dot{\theta }}_{l}=w_{l}\\ {\dot{w}}_{l}=\frac{k}{J_{l}}(\theta_{l}-\theta_{m})-\frac{mgh}{J_{l}}\sin(\theta_{l}) \end{array}\right.$$

where $\theta_{m}$ and $\theta_{l}$ are the angular rotations of the motor and link, and $w_{m}$ and $w_{l}$ are the angular velocities of the motor and link, respectively. $J_{m}$ and $J_{l}$ are the motor inertia and connecting rod inertia, respectively. k and $k_{\tau }$ are the torsional spring constant and amplifier gain, and G,m and h are the viscous friction coefficient, pointer mass, and link length, respectively. Let $x=[\theta_{m},w_{m},\theta_{l},0.1w_{l}{]}^{T}.$ (40) can be written in the form of (24), whose system matrices are given as follows:

(41) $$A=\left[\begin{array}{llll} 0 & \;\;\;\;\;\;1 & \;\;0 & \;\;\;\;\;\;0\\ -48.6 & \;\;-1.25 & \;\;48.6 & \;\;\;\;\;\;0\\ 0 & \;\;\;\;\;\;0 & \;\;0 & \;\;\;\;\;\;10\\ 1.95 & \;\;\;\;\;\;0 & -1.95 & \;\;0 \end{array}\right],\;B=\left[\begin{array}{l} 0\\ 21.6\\ 0\\ 0 \end{array}\right],\;\;C=\left[\begin{array}{c} 1\;\;\;0\;\;\;0\;\;\;0\\ 0\;\;\;1\;\;\;0\;\;\;0\\ 0\;\;\;0\;\;\;1\;\;\;0 \end{array}\right].$$

The distribution matrices of the disturbance and sensor fault are assumed to be

(42) $$B_{d}=\left[B_{d_{1}}\;\;B_{d_{2}}\right]=\left[\begin{array}{ll} 0.5 & \;\;\;0.2\\ 1 & \;\;\;0.3\\ 0.5 & \;\;\;\;\;0\\ 1 & -0.2 \end{array}\right],\;\;D=\left[\begin{array}{c} 1\\ 0\\ 0 \end{array}\right].$$

The control input signal is given as follows:

(43) $$u=-K_{y}y+0.5$$

where

(44) $$K_{y}=\left[0.05\;0.02\;0\right].$$

The unknown input disturbances are $d_{1}=sin(4\pi t)$ and $d_{2}=0.5sin(\pi t)$ and the initial state vector is given as $x\left(0\right)={\left[2\;\;\;40\;\;\;10\;\;\;-0.5\;\;\;\right]}^{T}$. The nonlinear term is:

(45) $$\psi \left(t,x\right)=\left[\begin{array}{c} 0\\ 0\\ 0\\ -0.333sin\left(x_{3}\right) \end{array}\right],\;\;$$

We consider different sensor faults under various intervals shown in Table 1, which can cover most of the existing faults in industrial practices including biased fault, incipient slope fault, measurement effectiveness loss, low-frequency sinusoidal fault, and high-frequency sinusoidal signal fault, and square wave signal fault.

*(i)*  *The proposed adaptive UIO estimation technique*

Choosing r=0.48, ε=40 and Γ=10 and using design procedure 2, one can obtain the following gains for the robotic manipulator:

$$M=\left[\begin{array}{ccc} 0.1667 & 0.3333 & 0.1667\\ 0.3333 & 0.6667 & 0.3333\\ 0.1667 & 0.3333 & 0.1667\\ 0.3333 & 0.6667 & 0.3333 \end{array}\right],\;T=\left[\begin{array}{cccc} 0.8333 & -0.3333 & -0.1667 & 0\\ -0.3333 & 0.3333 & -0.3333 & 0\\ -0.1667 & -0.3333 & 0.8333 & 0\\ -0.3333 & -0.6667 & -0.3333 & 1 \end{array}\right],$$

$$L_{1}=\left[\begin{array}{ccc} 136.1849 & -122.3783 & 184.5829\\ -18.7326 & 82.5755 & 24.8910\\ 22.2723 & -8.1410 & 97.7857\\ 9.2067 & -8.4649 & 214.8044 \end{array}\right],\;L_{2}=\left[\begin{array}{ccc} -12.8223 & -25.6004 & -12.8223\\ -29.9099 & -59.8278 & -29.9099\\ -14.4396 & -28.8655 & -14.4396\\ -35.4655 & -70.9080 & -35.4655 \end{array}\right],$$

$$L=L_{1}+L_{2}=\left[\begin{array}{ccc} 2.1864 & -0.8810 & 2.0071\\ -0.3438 & 9.2785 & 0.4179\\ 0.9955 & 0.4180 & 5.8298\\ -0.2849 & -1.8655 & 3.8907 \end{array}\right]\times 10^{3},$$

$$N=\left[\begin{array}{cccc} -119.9865 & 123.6282 & -200.7812 & -1.6670\\ 2.5343 & -83.3254 & -8.6926 & -3.3330\\ -6.0739 & \;8.3909 & -113.9841 & 8.3330\\ 25.1449 & 8.9650 & -249.1560 & -3.3330 \end{array}\right],$$

$$F=\left[2.6384\;\;\;0.5372\;-1.0113\right]\times 10^{3}.$$

*(ii)*  *The augmented UIO estimation technique [17]*

For comparison, the augmented UIO estimation technique in [17] is simulated here. Let

$\bar{x}=\left[\begin{array}{c} x\\ {\dot{f}}_{s}\\ f_{s} \end{array}\right]$, system (40) can be augmented to the following:

(46) $$\left\{\begin{array}{c} \dot{\bar{x}}\left(t\right)=\bar{A}x\left(t\right)+\bar{B}u\left(t\right)+{\bar{B}}_{d}d\left(t\right)+\bar{\psi }\left(t,x\right)\;\\ y\left(t\right)=\bar{C}x\left(t\right)\;\;\;\;\;\;\;\;\;\;\;\;\;\;\;\;\;\; \end{array}\right.$$

where

$$\bar{A}=\left[\begin{array}{ccc} A & 0 & 0\\ 0 & 0 & 0\\ 0 & I & 0 \end{array}\right],\;\bar{B}=\left[\begin{array}{c} B\\ 0\\ 0 \end{array}\right],{\bar{B}}_{d}=\left[\begin{array}{c} B_{d}\\ 0\\ 0 \end{array}\right],\;\bar{C}=\left[\begin{array}{ccc} C & 0 & D \end{array}\right],\bar{\psi }\left(t,x\right)=\left[\begin{array}{c} \psi \left(t,x\right)\\ 0\\ 0 \end{array}\right].$$

The nonlinear augmented UIO is given in the form of:

(47) $$\left\{\begin{array}{c} \dot{\bar{z}}\left(t\right)=\bar{R}\bar{z}\left(t\right)+\bar{T}\bar{B}u\left(t\right)+\bar{T}\bar{\psi }\left(t,x\right)+\bar{K}y(t)\;\\ \hat{\bar{x}}\left(t\right)=\bar{z}\left(t\right)+\bar{H}\;y(t)\; \end{array}\right.$$

Using Theorem 3 in literature [17] and choosing the index performance r=0.48 and parameter ε=40, one can obtain the following gains of the augmented observer:

$$\bar{H}=\left[\begin{array}{ccc} 0.1667 & 0.3333 & 0.1667\\ 0.3333 & 0.6667 & 0.3333\\ 0.1667 & 0.3333 & 0.1667\\ 0.3333 & 0.6667 & 0.3333\\ 0 & 0 & 0\\ 0 & 0 & 0 \end{array}\right],$$

$$\bar{T}=\left[\begin{array}{cccccc} 0.8333 & -0.3333 & -0.1667 & 0 & 0 & -0.1667\\ -0.3333 & 0.3333 & -0.3333 & 0 & 0 & -0.3333\\ -0.1667 & -0.3333 & 0.8333 & 0 & 0 & -0.1667\\ -0.3333 & -0.6667 & -0.3333 & 1 & 0 & -0.3333\\ 0 & 0 & 0 & 0 & 1 & 0\\ 0 & 0 & 0 & 0 & 0 & 1 \end{array}\right],$$

$$\bar{K}=\left[\begin{array}{ccc} 24.2880 & -21.8704 & 18.6186\\ -11.3007 & 3.6848 & -4.2346\\ 2.4013 & \;2.9126 & 8.9394\\ 7.5651 & -19.8579 & 26.4848\\ -5.8728 & 14.7601 & -23.6473\\ -4.6680 & 11.7462 & -18.8244 \end{array}\right],$$

$$\bar{R}=\left[\begin{array}{cccccc} \;-5.9437 & 27.4187 & -32.6710 & -1.6670 & -0.1667 & -22.1420\\ -7.3008 & -9.2433 & 18.0299 & -3.3330 & -0.3333 & 8.8976\\ 16.8727 & 3.4902 & -22.0621 & 8.3330 & -0.1667 & 0.6743\\ 27.4674 & \;21.7249 & -60.1555 & -3.3330 & -0.3333 & -6.8842\\ 4.7632 & -16.9837 & 22.5377 & 0 & 0 & 4.7632\\ \;3.6654 & -13.7550 & 17.8217 & 0 & 1 & 3.6654 \end{array}\right],$$

**Figure 6:**
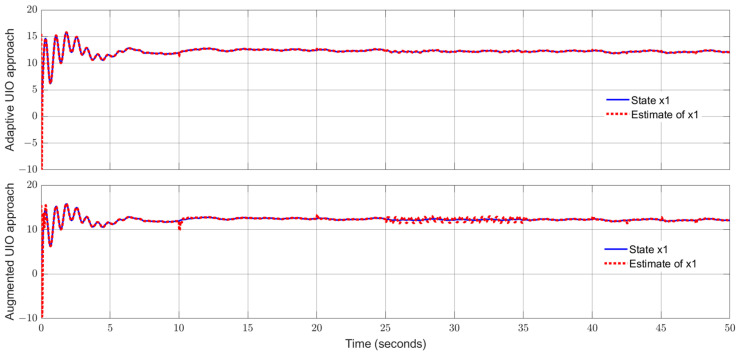
State $x_{1}$ (motor angular) and its estimate.

**Figure 7:**
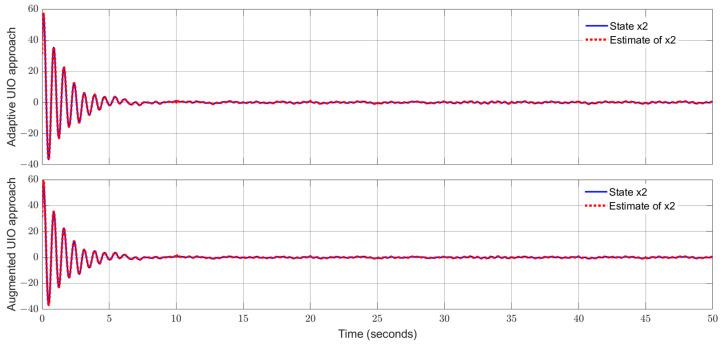
State $x_{2}\;$(motor angular velocity) and its estimate.

**Figure 8:**
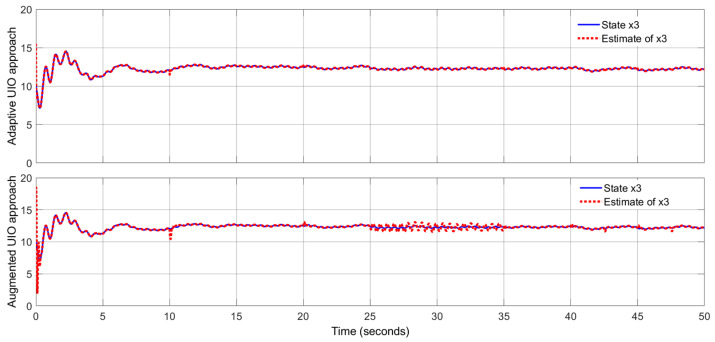
State $x_{3}$ (link angular) and its estimate.

**Figure 9:**
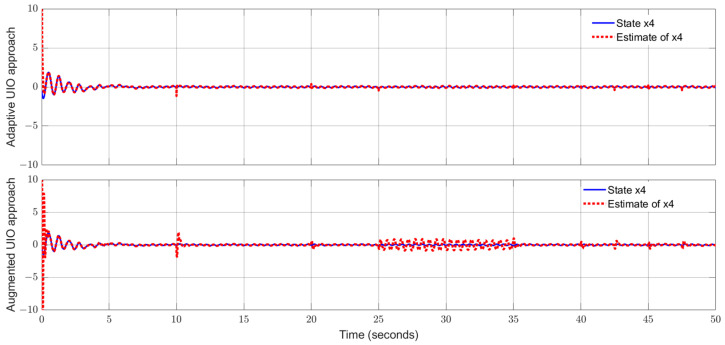
State $x_{4}$ (10% link angular velocity) and its estimate.

**Figure 10:**
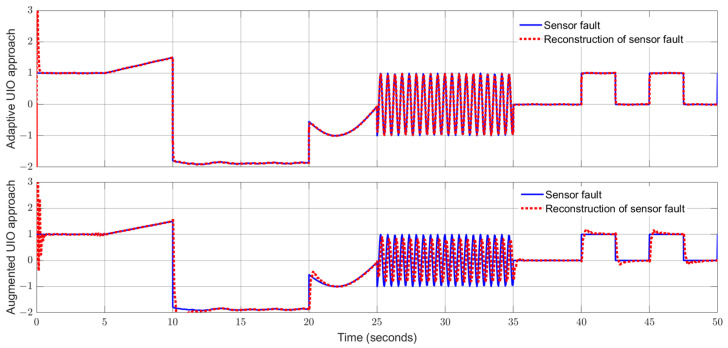
Sensor fault $f_{s}$ and its reconstruction.

Applying the proposed adaptive UIO estimator in the form of (6) and (26) and implementing the augmented UIO in the form of (47), one can obtain the curves of the states, sensor fault and their estimates shown in Figure 6, Figure 7, Figure 8, Figure 9 and Figure 10. Based on the simulations, we can have comparisons of the estimation performance of the proposed adaptive UIO technique and the existing augmented UIO approach in Table 2. From Table 2, one can see the proposed adaptive UIO estimation technique has a better tracking performance particularly when the fault is a high-frequency fault signal.

## 6. Conclusions

In this paper, a novel sensor fault estimator, that is, an adaptive UIO technique, was proposed by synthesizing an unknown input observer and adaptive observer. The robustness was ensured by decoupling input uncertainties using an unknown input observer and attenuating the un-decoupled disturbed signals via the linear matrix inequality approach. Both line dynamic systems and Lipschitz nonlinear dynamic systems were investigated. The proposed estimation algorithms were well-validated by two engineering-oriented systems. From the simulations, one can see the proposed adaptive UIO technique outperforms the existing augmented UIO approach when the sensor fault signal is high-frequent. In the future, it is of interest to investigate how adaptive observer techniques can be applied to reconstruct multiple sensor faults, which is under way.

## Figures and Tables

**Table 1:** Sensor fault under various intervals.

(1) $\mathrm{Biased}\;\mathrm{fault}:\;f_{s}=1,\;\;0\leq t<5$.
(2) $\mathrm{Incipient}\;\mathrm{fault}:\;f_{s}=0.1\left(t-5\right)+1,\;\;5\leq t<10$.
(3) $\mathrm{Measurement}\;\mathrm{effectiveness}\;\mathrm{loss}:\;f_{s}=-0.2y(t)$, 10≤t<20.
(4) $\mathrm{Low}-\mathrm{frequency}\;\sin\mathrm{usoidal}\;\mathrm{signal}\;\mathrm{fault}:\;f_{s}=\sin\left(0.5t\right),$ 20≤t<25.
(5) $\mathrm{High}-\mathrm{frequency}\;\sin\mathrm{usoidal}\;\mathrm{signal}\;\mathrm{fault}:\;f_{s}=\sin\left(12t\right),$ 35≤t<40.
(6) $\mathrm{Intermittent}\;\mathrm{fault}:\;f_{s}=$ square wave signal, t≥40.

**Table 2:** Performance comparison between adaptive UIO and augmented UIO.

Sensor Fault Type	Proposed Adaptive UIO	Existing Augmented UIO
Biased fault	Track well	Track well
Incipient fault	Track well	Track well
Measurement effectiveness loss	Work well with a quicker tracking	Track well
Low-frequency sinusoidal signal fault	Work well with a quicker tracking	Track well
High-frequency sinusoidal signal fault	Better tracking performance compared with the augmented UIO	Tracking performance is reduced with a high-frequency sensor fault signal
Intermittent square wave fault	Work well with a quicker tracking	Track well

## Data Availability

No new data were created in this study. Data sharing is not applicable to this article.
